# Determinants of AI Scribe Adoption in Allied Health: Mixed Methods Study Guided by the Capability, Opportunity, and Motivation Model for Behavior Change

**DOI:** 10.2196/88088

**Published:** 2026-10-15

**Authors:** Laura Ryan, Laetitia Hattingh, Dominic Wall, Hayley Stanich, Nicole Ross, Juan da Cal, EJ Milne, Rachel Wenke

**Affiliations:** 1 Gold Coast Health, 1 Hospital Blvd, Southport, Queensland, 4215, Australia, 61 56873041; 2 School of Pharmacy and Medical Sciences, Griffith University, Southport, Queensland, Australia; 3 School of Pharmacy and Pharmaceutical Sciences, The University of Queensland, Brisbane, Queensland, Australia; 4 Faculty of Health, Charles Darwin University, Darwin, Northern Territory, Australia; 5 School of Health Allied Health, Sport & Social Work, Griffith University, Southport, Queensland, Australia; 6 Faculty of Health Sciences and Medicine, Bond University, Gold Coast, Queensland, Australia

**Keywords:** artificial intelligence, AI, AI medical scribe, digital scribe, allied health, electronic medical record

## Abstract

**Background:**

AI scribes are emerging tools designed to reduce clinician documentation burden and improve clinical quality and efficiency by transcribing and documenting patient interactions in real-time. Despite growing evidence of their efficacy and impact across medical disciplines, there remains limited research on factors that influence successful implementation, particularly in allied health practice.

**Objective:**

This study explored the factors influencing the adoption of an AI scribe, including barriers and enablers among allied health clinicians, to inform ongoing implementation in public health settings.

**Methods:**

A mixed methods design was employed within a large Australian hospital and health service. Data were collected through surveys (n=97) and semistructured interviews (n=27) with allied health clinicians across multiple disciplines, including both adopters and nonadopters of an AI scribe. Survey data were analyzed descriptively. Interview transcripts were coded and thematically analyzed using a deductive approach guided by the COM-B (Capability, Opportunity, Motivation–Behavior) model to identify determinants of behavior.

**Results:**

Adoption was shaped by factors that could function as both barriers and enablers across the domains of capability, opportunity, and motivation. Enablers included tailored training, technology literacy, access to equipment and suitable recording spaces, leadership and peer support, and clear organizational communication. The absence of these factors, along with time and capacity constraints and unclear processes, hindered adoption. Responses to change, perceptions of the AI scribe’s value, safety, reliability, and privacy, and prior experience with similar tools also influenced adoption, depending on individual perspectives.

**Conclusions:**

The successful implementation of an AI scribe in allied health settings requires multifaceted strategies that address COM-B factors. Key priorities include allocating sufficient time for uptake and adoption, providing tailored training, ensuring access to relevant resources, fostering leadership and peer support, and providing organizational direction. These findings offer actionable insights to guide future implementation efforts and advance digital transformation in allied health practice.

## Introduction

AI scribes are digital tools designed to assist clinicians by transcribing and documenting patient interactions in real-time, thereby reducing the documentation burden and enhancing clinical efficiency [1,2]. These tools use natural language processing and machine learning to generate high-quality clinical notes, aiming to streamline workflows and enhance patient care [2-8].

The existing literature on AI scribes has predominantly focused on assessing their effectiveness, accuracy, and impact on patient care and clinician burnout [3,7,9-11]. Reported benefits include reduced documentation burden, improved efficiency, and enhanced clinical engagement, while potential risks relate to data privacy and security, transcription inaccuracies, and overreliance on technology [3,7,8,12-14]. Additionally, this research has been largely situated within medical disciplines [11,14-16]. While existing studies provide valuable insights into the potential of AI scribes to enhance clinical workflows and improve health care delivery in medical disciplines, there remains a significant gap in the literature regarding their application in allied health settings [17].

Allied health professionals make up about 20% of Australia’s health care workforce and represent a wide range of disciplines working across diverse clinical settings [18]. While research is limited, emerging evidence suggests that, as in medical disciplines, the use of AI scribes in allied health offers benefits such as reducing documentation burden, enhancing productivity, and improving both patient care and clinician well-being [17,19]. However, there is limited empirical research on the factors influencing adoption, including barriers and enablers to implementation. Given the diverse documentation needs and varied clinical environments within allied health, tailored strategies and supporting infrastructure may be essential for effective implementation of AI scribes [20-23].

A recent scoping review examining barriers to AI scribe adoption among medical disciplines identified several implementation challenges, including upfront costs, integration with existing systems, and time-intensive training requirements [24]. While these findings offer a relatively broad perspective, they do not provide a nuanced understanding of the individual-level factors that influence a clinician’s decision to adopt or reject AI scribe technology. There is increasing recognition of the need to develop thoughtful clinical protocols to support the safe and effective implementation of AI scribes [12]. However, without understanding the barriers and enablers to adoption, such protocols risk being misaligned with clinical practice.

To address this gap, this study investigates the implementation of an AI scribe within a large Australian public hospital and health service. To understand factors influencing the adoption of an AI scribe in allied health practice, this study draws on established behavior change theories. The COM-B model provides a foundational lens by conceptualizing Behavior as a function of capability, opportunity, and motivation[25]. The behavior change wheel was used to identify behavioral determinants and inform targeted strategies to support implementation. Together, these frameworks provide a structured approach to understanding drivers and barriers to integrating AI scribes into clinical workflows [25].

While the COM-B model has previously been used to understand AI adoption in hospital settings [26], this study is unique in that it has applied theoretical frameworks for behavior change to explore the adoption of an AI scribe within the allied health setting. Specifically, this study explored the barriers and enablers to AI scribe uptake among allied health clinicians to inform the design of targeted strategies to support effective and sustainable implementation in clinical settings. The following research question answered by this study is: What barriers and enablers influence clinician behavior in implementing the AI scribe regarding clinician capability, motivation and opportunity?

## Methods

### Methodological Approach

Guided by a pragmatic paradigm, emphasizing practical inquiry and the use of diverse methods to address real-world problems, this study used a convergent mixed-methods design. Survey and interview data were collected in parallel to examine how the AI scribe was integrated into clinical practice [27,28]. The survey findings offered valuable descriptive and contextual information, while the interviews provided richer, more detailed insights into participants’ experiences. Bringing these approaches together strengthened the credibility of the results and allowed for a more holistic understanding of the factors influencing acceptance [29]. A qualitative-dominant mixed-methods design was used (QUAL+quan), with qualitative data forming the primary strand and quantitative data used to complement and contextualize the findings [30]. The reporting of this study was guided by the MMR-RHS (Mixed Methods Reporting in Rehabilitation & Health Sciences) guidelines (Checklist 1).

### Setting

The study took place within a large Australian public hospital and health service located in a metropolitan region. This service employs around 1600 allied health professionals and delivers hospital and community services to a population exceeding 700,000 [31].

### Positionality of the Research Team

The research was undertaken by a multidisciplinary team that included hospital consumers, allied health clinicians, clinical leaders, researchers, and a representative from the hospital’s digital health unit, ensuring the study’s relevance and credibility. Consumer representatives were engaged through the health service’s Consumer Advisory Group (HS and EJM). One brought prior research experience, while the other was new to the process; both were compensated for their contributions. They were involved throughout the whole research life cycle and are named as coauthors. To further strengthen rigor, a reference group comprising consumers, clinicians, and health service and research leaders was convened to provide guidance and oversight at critical stages of the project. The interviews and analysis were conducted by an experienced hospital social worker and health services researcher with no strong pre-existing views on the AI scribe beyond the need for further study. Most participants were not previously known to the researchers; some were known professionally, but there were no close or personal relationships.

### Ethical Considerations

Ethical approval was obtained from Gold Coast Hospital and Health Service Human Research Ethics Committee (HREC/2025/QGC/118766). Research governance approval was given by the Gold Coast Hospital and Health Service (SSA/2025/QGC/118766). Participants were provided with an electronic information sheet via REDCap (Vanderbilt University) outlining the study, including the risks and benefits of participation. Participation was voluntary, and informed consent was obtained electronically through REDCap. No compensation was provided to the clinicians who participated in this study. All study data were deidentified for analysis. However, survey data for participants who provided their email addresses were identifiable to two of the investigators, who had access to the raw data extracted from REDCap, prior to analyses.

### Participants and Recruitment

Eligible participants were allied health clinicians from professions including physiotherapy, occupational therapy, social work, dietetics, speech pathology, pharmacy, psychology, audiology, and podiatry with over 2 years of clinical experience. The decision to exclude participants with less than 2 years of experience was made because some would have been ineligible to use the AI scribe due to permissions set by some areas of the health service, and because the study aimed to capture the perspectives of more established clinicians. Although HP6 and HP7 classifications were included as response options in the survey demographics to enable participation from clinicians in senior leadership roles, no participants were recruited from these grades. At the commencement of data collection, approximately 280 allied health clinicians had been granted access to the AI scribe. A convenience sampling approach was used for the clinician survey, while purposive sampling guided the recruitment of interviewees to ensure representation across professions. Both adopters and nonadopters were included to provide a comprehensive understanding of the factors influencing adoption. Invitations to participate were distributed via email, team meetings, and targeted outreach informed by AI scribe usage data.

### Data Collection

Quantitative and qualitative data collection was undertaken concurrently between August 2025 and October 2025.

#### Survey

Clinician surveys were administered via REDCap over a 6-week period to capture experiences and perceptions of AI scribe adoption. The survey design was informed by both the technology acceptance model and the COM-B framework (Multimedia Appendix 1) [25,32]. The present paper concentrates on COM-B factors influencing adoption and integration [25].

All survey respondents answered 5 demographic questions. Nonadopters responded to a broader set of items, including a Likert-scale question and several open-ended questions exploring adoption-related factors. Adopters completed 3 Likert-scale items and 5 open-ended questions about their experiences and perceptions of the AI scribe, along with 5 demographic and usage questions. The survey was piloted with both adopters and nonadopters, resulting in minor adjustments to the question order and wording before its final use.

#### Interviews

To gain insights into experiences and contextual factors, semistructured interviews were conducted via Microsoft Teams. Interested participants received an electronic consent form via REDCap, and demographic data were collected upon completion. Interviews lasted between 15 and 60 minutes. Two tailored interview guides were developed and piloted, one for clinician adopters and another for nonadopters (Multimedia Appendices 2 and 3, respectively). Guides explored experiences and determinants of adoption, informed by the COM-B model [25].

All interviews were scheduled at the participants’ convenience, conducted by a member of the research team (LR), and recorded via Microsoft Teams. Recordings were transcribed in Microsoft Teams, and the transcripts were verified against the audio by LR, with identifiers removed and unique participant IDs assigned.

### Data Analysis

Survey data were exported from REDCap into Microsoft Excel. Descriptive statistics (frequencies and percentages) were used to summarize closed-ended items. Responses from the 5-point Likert scale were dichotomized for analysis. “Agree” and “Strongly Agree” were combined to represent overall agreement, while “Disagree” and “Strongly Disagree” were combined to represent overall disagreement, and percentages were reported accordingly. Neutral responses were not grouped with agreement or disagreement responses but were instead treated as a separate category. Survey items, including reverse-worded statements, were analyzed using their original coding, with no scale-level reverse scoring applied, as responses were used for descriptive and comparative analyses alongside qualitative findings.

Interview transcripts were coded and themed in NVivo (Lumivero), with a deductive approach guided by COM-B to identify determinants of behavior [25,33]. Initial coding was completed by 1 researcher (LR), who then grouped codes into potential themes aligned with COM-B domains. These themes were reviewed and refined through discussion with the wider research team to resolve discrepancies and ensure interpretive rigor. Finally, these were considered in relation to the behavior change wheel to identify potential intervention functions and policy categories relevant to supporting AI scribe adoption [25]. A pragmatic approach was taken, recognizing that some strategies identified by participants did not align neatly with the ehavior change wheel and were therefore incorporated flexibly to ensure practical applicability.

Data synthesis adopted a qualitatively driven mixed-methods design, prioritizing qualitative findings for their depth and contextual richness relative to survey data. Interviews offered detailed insights into experiences and perceptions, while survey data provided complementary breadth and supported triangulation of key themes. Qualitative data from interviews were first analyzed to establish a foundational framework of themes. Subsequently, quantitative survey data were analyzed and mapped against these emergent qualitative categories. This mapping process confirmed a high degree of alignment between the 2 datasets, allowing for the integration of descriptive statistics to validate and illustrate the prevalence of the qualitatively identified themes. This approach reflects best practice for qualitatively led mixed-methods research, where qualitative evidence serves as the primary interpretive lens [30]. Open-ended survey responses were reviewed alongside interview data to check for consistency; however, they did not generate additional unique insights and are therefore not presented here.

## Results

### Participant Characteristics and Overview of Results

The study included 27 clinician interviews across multiple allied health disciplines and 110 survey submissions. Of these, 97 surveys were analyzed. Eleven were excluded due to substantial incompleteness (consent or demographic data only), and two were excluded for not meeting the eligibility criteria (not allied health clinicians). To ensure a comprehensive representation of perspectives, partially completed surveys were retained. Of the 97 surveys analyzed, 58 were completed by adopters of the AI scribe, and 39 were nonadopters. For the interviews, 22 participants were adopters, and 5 were nonadopters. Further demographic characteristics of participants are summarized in Table 1.

**Table 1.** Demographic information on survey and interview participants^a^.

Category and component	Survey (n=97), n (%)	Interview (n=27), n
AI scribe use		
Adopters	58 (60)	22
Nonadopters	39 (40)	5
Disciplines		
Physiotherapy	20 (21)	5
Social work	17 (18)	5
Psychology	12 (12)	4
Occupational therapy	11 (11)	3
Speech pathology	11 (11)	3
Dietetics	9 (9)	3
Pharmacy	8 (8)	2
Audiology	6 (6)	1
Podiatry or other	3 (3)	1
Clinical settings of AI scribe use		
Inpatient	6 (6)	—^b^
Outpatient or community health	49 (51)	20
Emergency department	2 (2)	1
Other including nonadopters	42 (43)	6
Role level		
HP3 (entry-level clinician)	29 (30)	8
HP4 (senior)	42 (43)	10
HP5 (team leader or advanced)	26 (27)	9
Years in practice		
2‐5	16 (16)	6
6‐10	19 (20)	6
11‐19	32 (33)	8
20+	30 (31)	7

a Participants could select multiple clinical settings.

b Not applicable.

Analysis revealed an interchange among COM-B domains that shapes adoption and integration into practice. Qualitative data from clinician interviews are presented as the primary narrative, and survey findings are integrated throughout to reinforce and elaborate on key themes. Figure 1 outlines the key determinants, while Table 2 summarizes these under the COM-B components, indicating whether each acted as a barrier or enabler. Findings are reported in relation to the COM-B model. Potential intervention strategies derived from the behavior change wheel mapping are presented in the “Discussion” section [25].

**Figure 1.**
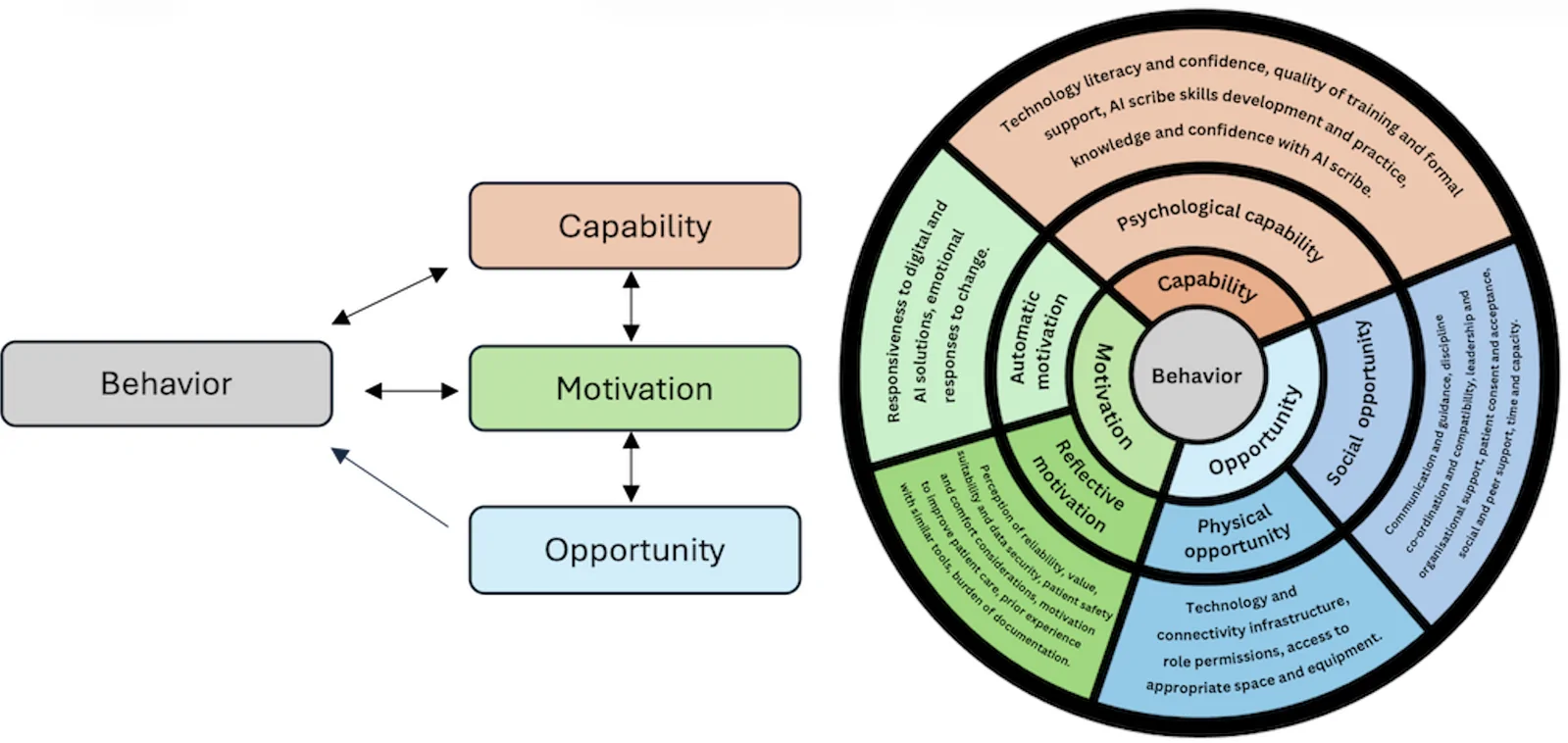
Visualization of qualitative and quantitative themes across COM-B (Capability, Opportunity, Motivation–Behavior) domains for AI Scribe use among allied health clinicians.

**Table 2.** Mapping of qualitative and quantitative determinants of allied health clinicians’ AI scribe adoption to COM-B (Capability, Opportunity, Motivation–Behavior) framework.

COM-B component and themes from data		Enabler	Barrier	Comments
Psychological capability				
Quality of training and formal support		✓	✓	Enabler when present and a barrier when absent
Knowledge and confidence in using the AI scribe		✓	✓	Enabler when present and a barrier when absent
AI scribe skills development and practice		✓	✓	Enabler when present and a barrier when absent
Technology literacy and confidence		✓	✓	Mainly an enabler, although absence occasionally posed a barrier
Physical opportunity				
Role permissions			✓	Barrier
Technology and connectivity infrastructure			✓	Barrier
Access to appropriate equipment or suitable spaces for use		✓	✓	Enabler when present and a barrier when absent
Social opportunity				
Communication and guidance or clarity of processes		✓	✓	Enabler when present and a barrier when absent
Discipline coordination and compatibility		✓	✓	Enabler when present/compatible and a barrier when absent/incompatible
Leadership and organizational support		✓	✓	Enabler when present and a barrier when absent
Patient consent and acceptance		✓	✓	Mainly an enabler; consent-related factors occasionally posed a barrier
Social and peer support		✓	✓	Enabler when present and a barrier when absent
Time and capacity among competing priorities		✓	✓	Enabler when present and a barrier when absent
Reflective motivation				
Perception of reliability, value, and appropriateness		✓	✓	Acts as enabler or barrier depending on perception
Perception of data security		✓	✓	Acts as enabler or barrier depending on perception
Motivation to improve patient care		✓		Enabler
Consideration of patient safety and comfort		✓	✓	Acts as enabler or barrier depending on perception
Prior experience with similar tools		✓		Enabler
Perceived burden of traditional documentation		✓	✓	Mainly an enabler; can act as barrier depending on perception
Automatic motivation				
Responsiveness to digital and AI solutions		✓	✓	Mainly an enabler; can act as barrier depending on perception
Emotional responses to change		✓	✓	Acts as enabler or barrier depending on perception

### Psychological Capability

#### Quality of Training and Formal Support

Participants highlighted the need for both cognitive and practical skills for effective AI scribe use, with training quality and format being central. All but one of the interviewed adopters had completed the health service’s organization-wide online training module, which most viewed as a useful introduction. Survey data supported this, with 69% (n=40) agreeing it prepared them to use the AI scribe. Participants highlighted the value of offering training through multiple formats (eg, webinars, Q&A sessions, and in-person demonstrations) and providing discipline-specific training and resources, such as videos featuring allied health examples, “I think being trained by a speech pathologist, like being trained by someone from your profession that understands the subject matter, will probably be really helpful too” (P10). Some participants also valued additional formal support, such as individualized assistance and troubleshooting. Notably, in the survey, 74.4% (n=29) of nonadopters reported being offered the online training, indicating that access alone did not necessarily lead to adoption. Multimedia Appendix 4 presents survey data for adopters, and Figure 2 presents data on nonadopters.

**Figure 2.**
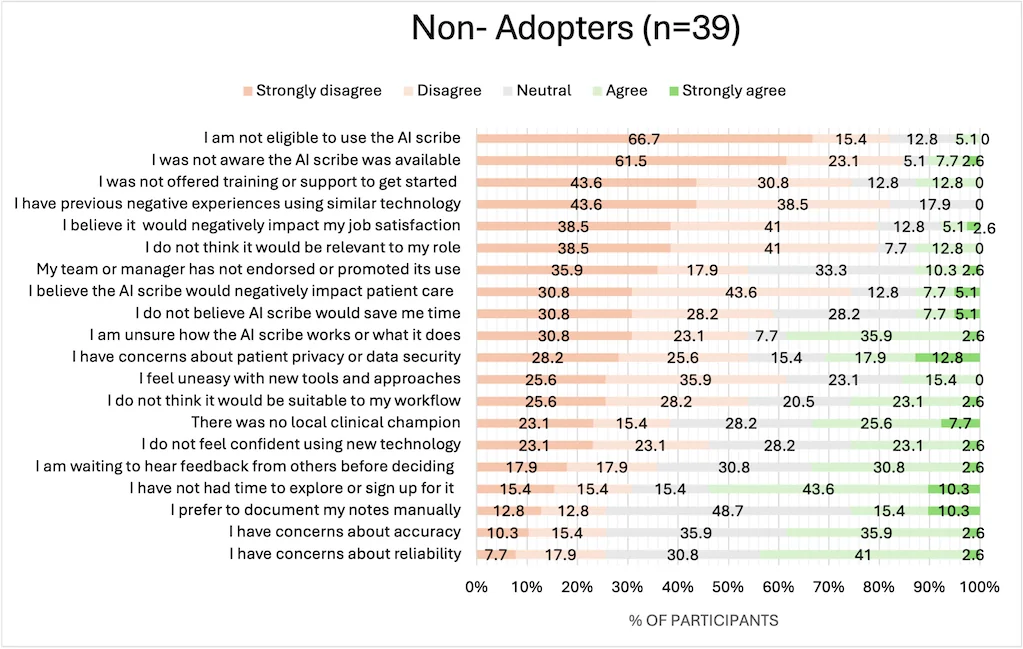
Survey results on AI scribe implementation among allied health nonadopters.

#### Knowledge and Confidence in Using the AI Scribe

A basic understanding of the AI scribe’s functions and confidence in its use enabled integration, “I understand generally what it is and what its functions are.” (P12). Survey data supported this, with most adopters reporting they understood what an AI scribe is (n=57, 98.3%) and how information was stored (n=53, 91.4%). In contrast, gaps in understanding appeared to hinder uptake. As 1 nonadopter interview participant noted:

*I’m going to be using this headset, and it would be on the same computer. So those kind of things, I think that will be the barrier…I don’t know how it looks like.*

[P8]

This uncertainty was echoed in the survey, where 38.5% (n=15) of nonadopters reported being unsure about how the AI scribe works or what it does.

#### AI Scribe Skills Development and Practice

Skill development, such as remembering to activate the scribe and adapting communication styles, was also critical, “I have to be conscious to verbalize things in a particular way for it to capture it in the right way” (P25). Opportunities to practice using the AI scribe helped build skills and confidence, which, in turn, supported adoption, “I invested that time and effort, practiced it a bit, then I actually think it’s really quite easy” (P14).

#### Technology Literacy and Confidence

Technology literacy and confidence facilitated adoption, “I feel like I’m able to kind of figure out apps pretty easily” (P3). However, a lack of digital skills was a barrier for some, “I’m a bit worried, I’m not tech-savvy in that regard” (P17). The survey further supported the role of digital literacy in facilitating AI scribe adoption, as 25.6% (n=10) of nonadopters lacked confidence in using new technology.

### Physical Opportunity

#### Role Permissions

Within the hospital, there were specific areas where clinicians were either permitted or prohibited from using the AI scribe. For example, in some areas, its use was not allowed until the team had investigated its suitability, “I need to get the go-ahead from the team leader to be one of those few people that kind of does all the, you know, how would this maybe work for the team?” (P17). In some areas, clinicians reported that they could only use the AI scribe if they met certain conditions, such as having over 2 years of clinical experience and access to a suitable recording space. In the survey, among nonadopters, 5.1% (n=2) felt ineligible, and 12.8% (n=5) were neutral about their eligibility, suggesting that perceived ineligibility was a barrier for some clinicians and that uncertainty about permissions may have influenced uptake.

#### Technology Infrastructure, Access to Appropriate Equipment and Suitable Spaces

Practical and technical conditions significantly influenced adoption. Reliable technology infrastructure, appropriate equipment, and environments facilitated effective use of the AI scribe, while their absence—and occasional technical failures—created barriers. For example, poor connectivity hindered trialing, “A lot of the places we go don’t always have great reception, so that has been a barrier to me trialing it.” (P1). Similarly, access to supportive resources, such as microphones, improved usability and therefore integration:

*We have those little mini microphones that we use with it. So, I think that’s been helpful to have, to make sure we’re getting the accurate recording.*

[P27]

The survey findings echoed these qualitative insights, with 81% (n=47) of adopters reporting access to a suitable environment for using the AI scribe, and only 3.4% (n=2) indicating they lacked the necessary equipment.

### Social Opportunity

#### Communication, Guidance, and Clarity of Processes

Social and organizational factors were influential. Clear communication and guidance about permissions, procedures, and legislation were seen as facilitators while their absence hindered adoption, “I don’t know if there is anything like the processes or procedures to utilize this software, so I don’t know if there are certain things that I can’t do with it” (P8).

#### Discipline Coordination and Compatibility

Perceptions of how well the AI scribe aligned with disciplinary requirements and standards of practice also shaped adoption. Where clinicians felt the AI scribe complemented their professional role, and where departments had progressed toward integrating it into routine workflows, uptake occurred:

*Is it within our own ethical code to be able to use this? I think there was initial uncertainty…but quickly that dissipated.*

[P14]

Conversely, the lack of departmental approaches to integration limited adoption, “This would be much better if there was a coordinated approach...because what’s the point of us all, we all use the same very similar documentation templates” (P10).

#### Leadership and Organizational Support

Leadership and organizational encouragement were noted as potential facilitators during interviews, with a few participants viewing leadership direction as crucial to uptake, “What will work for me and what is working for me is pressure from above” (P23). However, survey data presented a more complex picture. Among adopters, 63.8% (n=37) reported having a manager who encouraged them to try the AI scribe. However, a comparable proportion of nonadopters (n=21, 53.8%) also indicated managerial support, suggesting that other factors may have played a more decisive role in influencing adoption.

#### Patient Consent and Acceptance

Patient consent and acceptance were generally seen as enablers, as participants perceived a high level of patient acceptance or consent via indifference toward the use of the AI scribe. For example, 1 participant working in a setting where the AI scribe was used by multiple clinicians, and where patients encountered and had the AI scribe explained multiple times during a single clinic visit, stated:

*And a lot of our clinicians are using it now. So even if they haven’t seen me before, before I even get to explain it, they go,* “*yeah, yeah, yeah, that’s fine.”*

[P20]

In the survey, a total of 84.5% (n=49) of adopters agreed that obtaining patient consent was easy. However, some participants felt the consent process could be burdensome (eg, having to obtain permission for the AI scribe at every encounter with the same patient) or posed challenges, which acted as a barrier to use, “I do still feel quite uncomfortable asking patients if they’re happy for me to use it.” (P21).

#### Social and Peer Support

For adopters, peer networks offered encouragement to get started, as well as informal support and resources (such as shared templates), which further promoted uptake:

*I think my colleagues are using it too, everybody kind of being supportive and talking about it. “Oh, if you’ve got any problems, just let me know.”*

[P25]

Survey data reinforced this facilitator, with most adopters (n=50, 86.2%) agreeing that their colleagues were supportive of using the AI scribe, and one-third of nonadopters (n=13, 33.3%) indicating they were waiting for feedback from others before deciding whether to use it.

#### Time and Capacity Among Competing Priorities

The interviews highlighted that time and capacity amidst competing priorities were major barriers to adoption and full integration of the AI scribe, “That main barrier is just feeling like they’ve got the headspace to get started with it, the time and the headspace among many other competing demands” (P24). This was corroborated by survey data, with lack of time emerging as the most commonly reported barrier—53.8% (n=21) of nonadopters indicated they did not have sufficient time to explore or sign up for the AI scribe.

### Reflective Motivation

#### Perception of Reliability, Value, and Appropriateness

Reflective motivation was influenced by clinicians’ perceptions of the AI scribe’s reliability, value to their practice (including perceptions of accuracy), and its appropriateness to their role, “I mean, one initial barrier that kind of put me off, but it’s been resolved now, was not being able to use it for telehealth” (P16). Survey responses from nonadopters highlighted concerns about the AI scribe’s performance, with 38.5% (n=15) citing issues with accuracy and 43.6% (n=17) expressing doubts about reliability, reinforcing these factors as barriers to uptake.

#### Perception of Data Security

Confidence in patient privacy and data security was also identified as an enabler for use, “There is virtually no way that I am at all scared that my patients’ privacy will be impacted” (P11). Consistent with this, survey results showed almost all adopters (n=54, 93.1%) had no concerns regarding patient privacy. Conversely, 30.8% (n=12) of nonadopters reported concerns about privacy or data security, highlighting these factors as potential barriers to adoption for those not actively using the AI scribe.

#### Patient Care: Motivations and Impact

The drive to improve patient care was a strong motivator, “It provided me the opportunity to be more one-on-one in the moment with patients … and actually spend a bit more time just talking to them” (P5). However, perceptions of the AI scribe’s impact on patient safety and comfort sometimes acted as a barrier to initial uptake or full integration into clinical practice, “Particularly a lot of the most common ones are going to be delusions around people listening around devices. …if that is their presentation, I’ll probably opt to just not do it” (P16). Survey data provided some support for this, with 12.8% (n=5) of nonadopters reporting concerns about the AI scribe’s impact on patient care. Given the low proportion, this appeared to be a relatively minor barrier to initial adoption. Additionally, clinician observations of patient comfort also acted as a facilitator, helping overcome initial hesitations:

*I do also see a lot of patients with eating disorders… I was concerned that they won’t feel open or comfortable sharing with me everything that they would normally share in a consult. So, I was a little bit selective with who I asked to use AI scribe with initially, but it actually hasn’t been a barrier to using it, so I think it was my own perceived thoughts around it.*

[P1]

#### Prior Experience With Similar Tools and the Burden of Traditional Documentation

Prior experience with similar tools enhanced confidence in the AI scribe’s ability to perform effectively, supporting adoption:

*I’ve used other dictation software for clinical documentation and for letters. So, I knew that it was beneficial in that regard.*

[P18]

Additionally, the burden of traditional documentation heightened the appeal of the AI scribe, “I hate writing notes, and I’m a terrible writer, and I struggle to write, so this was like the ultimate fix to that” (P2). In contrast, among nonadopters, 25.6% (n=10) of survey respondents preferred to type their notes manually and 48.7% (n=19) were neutral.

### Automatic Motivation

#### Responsiveness to Digital and AI Solutions

Emotional responses played a role in the adoption of the AI scribe. Enthusiasm and curiosity about digital or AI solutions encouraged adoption, “I’d like to be across the opportunity to learn the newest technology and then make sure that we’re accessing it and in a way that supports our practice” (P7), while anxiety and skepticism acted as barriers, “It’s the AI and it’s probably a bit of a red flag” (P4).

#### Emotional Responses to Change

Openness to change appeared to facilitate AI scribe adoption, “I’m not very set in my ways, and I think there is always space for improvement” (P8). In contrast, some participants expressed resistance, “Everyone tells me it’s valuable and how I can use it, but emotionally I’m like…this is something new” (P24). Others acknowledged the need to evolve despite discomfort, “I’m a creature of habit, and I have to admit I’m not always keen to change, but I felt like we needed to move forward, because…otherwise you’ll get left behind” (P1). Survey data suggested that resistance to change was not a major barrier, with only 15.4% (n=6) of nonadopters reporting unease with new tools or approaches.

## Discussion

### Overview

This study examined the factors influencing the adoption and integration of an AI scribe among allied health clinicians in an Australian public health service, utilizing the COM-B model to guide the analysis. Findings revealed a delicate interplay between COM-B domains, with multiple determinants acting as barriers, enablers or both to implementation. Although research on AI scribe implementation remains limited, some findings align with existing literature—for example, the need for dedicated time to support adoption and training—which also emerged as an important finding in this study [24].

Building on earlier research exploring clinician perceptions of AI implementation [26], our study provides more nuanced insights specific to the implementation of AI scribe technology. While similar themes emerged, such as the need for leadership support, time and capacity constraints, and targeted education and training, unlike in Hoffman et al [26], concern about role replacement was not noted as a barrier to use, and apprehension about AI in health care was only a minor barrier. Instead, adoption appeared to be more strongly influenced by practical challenges associated with integrating the AI scribe into routine practice, such as having the necessary time, means (eg, suitable equipment), knowledge, skills, support, and organizational endorsement to initiate its use. This may reflect that most participants in our study were already adopters of the AI scribe. Alternatively, it may be because many participants recognized its potential usefulness and were instead grappling with its integration into practice. This highlights that, while broader understandings of AI in health care provide an important backdrop, more nuanced studies are needed to examine how specific technologies are implemented within real-world contexts [12].

Concerns regarding the processes of obtaining patient consent during encounters using the AI scribe, including the time burden, have been previously identified within the hospital outpatient setting, with clinicians identifying competing priorities during short appointment times as a challenge [34]. Lawrence et al [34 p.11] suggest shifting preliminary consent-related conversations to “earlier touchpoints in the patient journey,” such as at a reception desk or via a digitally enabled patient portal, and then having a secondary consent conversation with clinicians regarding consent [34]. Further exploration of these strategies and others that help obtain true informed consent when using AI scribes is important to minimize any ethical risk of assumed or poorly understood consent.

### Implementation Strategies Arising From Findings

Findings were mapped to the behavior change wheel to identify the intervention functions most relevant to supporting AI scribe adoption, as shown in Table 3. This process enabled the translation of behavioral determinants into actionable implementation strategies, highlighting areas where targeted support may facilitate uptake and sustained use (Textbox 1).

**Table 3.** Mapping of identified determinants of allied health clinicians’ adoption of an AI scribe to potential intervention functions and implementation strategies

COM-B^a^ component	Identified barrier or enabler	Targeted intervention functions	Potential implementation strategies
Psychological capability	Quality of training and formal support AI scribe skills development and practice	Education Training	Develop discipline-specific learning and induction modules Provide different modalities of training such as hands-on demonstrations, access to individual formal support, encourage regular practice
Physical opportunity	Technology and connectivity infrastructure Access to appropriate	Environmental restructuring Enablement	Ensure reliable hardware and connectivity Where appropriate, provide access to suitable spaces, equipment, or suitable spaces for use
Social opportunity	Time and capacity among competing priorities Communication and guidance or clarity of processes Social and peer support Discipline coordination and compatibility Leadership and organizational support Patient consent and acceptance	Modeling Enablement Environmental restructuring	Allocate time for uptake and integration Provide clear policy and guidance Identify and promote peer networks Consider a coordinated approach to adoption within each discipline (including discipline champions) Secure visible leadership and organizational endorsement Enable template sharing Provide information to patients
Reflective motivation	Perception of reliability, value, and appropriateness Perception of data security	Persuasion Education	Present evidence of improved workflow and documentation quality Address AI scribe concerns about reliability, accuracy, and data security
Automatic motivation	Emotional responses to change	Incentivization Enablement	Share success stories (eg, adopter and patient testimonials) Create safe trial environments with constructive feedback Normalize gradual uptake

a COM-B: Capability, Opportunity, Motivation—Behavior.

Textbox 1. **A summary of key intervention areas.**

**Capability**

- Tailored, discipline-specific education and training that build both technical and clinical confidence.
- Interventions to incorporate ongoing formal individualized support to reinforce skill development and encouragement of continued practice.

**Opportunity**

- Ensuring access to reliable internet connectivity, appropriate devices, and equipment.
- Providing access to suitable recording spaces was recommended, although some clinicians reported using the AI scribe in busy, open areas such as emergency departments.
- Allocating dedicated time for uptake and integration.
- Peer mentoring, including the sharing of resources such as templates and the potential use of discipline champions.
- Strong leadership endorsement, alongside establishing clear organizational policy and guidance regarding the use of the AI scribe.
- Providing clear information for patients to support the consent process.

**Motivation**

- Addressing concerns about accuracy, reliability, and privacy through transparent communication and evidence-based reassurance.
- Varied emotional responses such as curiosity and anxiety should be acknowledged and supported.
- Incorporate mechanisms for positive reinforcement, such as feedback loops and recognition of early adopters, and patient testimonials.

Together, these findings highlight that successful AI scribe implementation in allied health may depend on practical strategies that prioritize both Capability and Opportunity through reliable infrastructure, dedicated time, and access to tailored training and support, while understanding the underlying individual motivational factors that influence adoption, including the burden of traditional documentation practices, prior digital experience and perceptions of AI safety and reliability. Thus, effective implementation of AI scribe requires addressing both system-level factors (eg, training, infrastructure, and resourcing) while also acknowledging and supporting individual factors and attitudes across different experiences and perceptions that shape adoption and use. For example, a greater proportion of nonadopters had concerns related to privacy compared to those who had adopted the AI scribe. As such, providing targeted education to clinicians may alleviate some of these concerns, thereby addressing this barrier to adoption. Fostering supportive social, organizational, and emotional environments may also be critical for engaging nonadopters and promoting sustained, confident use. Addressing these tangible requirements can provide a strong foundation for effective and enduring adoption of AI scribes across diverse allied health settings.

### Strengths and Limitations

As a qualitatively dominant mixed-methods study, interviews provided depth and nuanced insight into clinicians’ and patients’ experiences with the AI scribe, while the survey offered broader contextual understanding and supported the wider relevance of emerging themes. A key strength of the study was the diversity of allied health clinicians involved, which enabled the capture of varied perspectives and enhanced the applicability of the findings.

Some limitations were also evident. Although the survey included clinicians from multiple disciplines, the number of respondents was small relative to the approximately 1600 allied health staff employed by a single Australian health service. While demographic comparisons were not completed, the findings may not fully reflect the wider workforce. Further research is also needed to better understand the perspectives and experiences of staff with less than two years of experience, as these were not a focus of the current study. Future research may also wish to recruit from multiple sites in different geographical areas to enhance the generalization of findings.

A further limitation was the use of a bespoke survey instrument that did not undergo formal psychometric validation. While the survey was theoretically informed by the COM-B and piloted with staff to maximize content validity, including a more formal validation of survey tools in future research may increase reliability and generalizability of the findings. Also, the survey design required respondents who had not completed AI scribe training to provide a response to a training-related item, with a neutral option available to reflect their lack of experience. However, as no untrained participants reported using the AI scribe with patients, this is unlikely to have influenced the study findings. Finally, while rigor was ensured through reflexive engagement with the data and regular discussion with the research team, we acknowledge that the use of multiple coders may have further enhanced the credibility of the qualitative analyses undertaken.

### Conclusions

This study advances understanding of AI scribe adoption by demonstrating how behavioral drivers across capability, opportunity, and motivation shape implementation. Practical barriers at the system level, particularly time and capacity constraints, were prominent, while enablers included high-quality training, peer and leadership support, clear communication, and access to appropriate resources. Individual motivational factors, such as perceptions of the AI scribe’s value, safety, reliability, and openness to change, as well as attitudes toward AI and digital solutions, also influenced uptake. Building on these insights, the paper discusses targeted interventions to help health services embed AI scribes into allied health practice at both the system and individual levels, thereby unlocking the potential of this promising technology.

## Supplementary material

10.2196/88088 Multimedia Appendix 1 Clinician survey.

10.2196/88088 Multimedia Appendix 2 Interview guide: adopter clinicians.

10.2196/88088 Multimedia Appendix 3 Interview guide: nonadopter clinicians.

10.2196/88088 Multimedia Appendix 4 Adopter survey results.

10.2196/88088 Checklist 1 MMR-RHS checklist.

## Abbreviations

COM-B: Capability, Opportunity, Motivation–Behavior
MMR-RHS: Mixed Methods Reporting in Rehabilitation & Health Sciences)
