# Supplementary material for: Determinants of AI Scribe Adoption in Allied Health: Mixed Methods Study Guided by the Capability, Opportunity, and Motivation Model for Behavior Change
Source: J Med Internet Res. 2026 Oct 15;28:e88088. doi: 10.2196/88088 (PMC13670898; doi:10.2196/88088)
Supplement: Supplementary file 2 [file jmir-v28-e88088-s001.docx]

# Clinician Survey: AI Medical Scribe Evaluation

**Information about this survey**

You are invited to take part in a research study exploring the use of an AI medical scribe by allied health clinicians at this health service. The AI scribe transcribes clinical conversations in real-time to assist with documentation. You have been invited to participate in this study because, as an allied health clinician, you may be eligible to use the AI scribe.

This survey will ask about your opinions about the AI scribe. It should take approximately 15-20 minutes to complete. You do **not** need to have used the AI scribe to participate.

**This study aims to understand different points of view, including why some people choose not to use the technology. All opinions are important and will help us learn more.**

Participation is **voluntary**. Your responses are **confidential,** and no identifying information will be shared.

All survey responses will be securely stored in REDCap, a secure data management platform.

**Do you consent to this survey?**

- **Yes**
- **No**

**Clinician Survey**

Thank you for agreeing to participate in this survey.

## Section 1: Demographic and Background

1. **Role:**
   - Dietitian
   - Occupational Therapist
   - Pharmacist
   - Physiotherapist
   - Podiatrist
   - Psychologist
   - Social Worker
   - Speech Pathologist
   - Other________________________________

2. **In what clinical settings do you use the AI scribe?**

- Outpatient clinic appointments
- Inpatient ward consultations
- Emergency department consultations
- Other (please specify): _______________
- I do not use the AI scribe

3. **HP Level:**

- HP3
- HP4
- HP5
- HP6
- HP7

4. **Years of experience in an allied health role:**
   - 2-5 years
   - 6-10 years
   - 11 years -19 years
   - 20 years +

**5. Which of the following best describes your experience with the AI scribe?**
(Please select one)

- I have completed the AI scribe online learning module and used the AI scribe with patients. *(Trained/Untrained Adopters Pathway)*
- I have **not** completed the AI scribe online learning module but have used the AI scribe with patients. *(Trained/Untrained Adopters Pathway)*
- I have completed the AI Scribe online learning module but have **not** used the AI scribe with patients. (*Non-Adopters Pathway*)
- I have **not** completed the AI Scribe online learning module and have **not** used the AI scribe. (*Non-Adopters Pathway*)

## *Two versions of Sections 2 and 3 are included to accommodate the survey's branching logic, with respondents completing only the section applicable to their responses.*

## ***** Non-Adopters Pathway*****

## Section 2: Barriers to training and adoption

1. **Instructions:** For each statement below, please indicate your level of agreement in relation to the AI Scribe:

1 = Strongly Disagree, 2 = Disagree, 3 = Neutral, 4 = Agree, 5 = Strongly Agree

a) I am not eligible to use the AI scribe (e.g., I am not >HP3.3 or do not have access to a private consultation room).
b) I was not aware the AI scribe was available.
c) I have not had time to explore or sign up for it.
d) I was not offered training or support to get started.
e) I am unsure how the AI scribe works or what it does.
f) I prefer to document my notes manually.
g) I do not feel confident using new technology.
h) I do not think it would be suitable to my workflow.
i) I do not think it would be relevant to my role.
j) I believe the AI scribe would negatively impact my job satisfaction.
k) I believe the AI scribe would negatively impact patient care.
l) I have concerns about accuracy.
m) I have concerns about reliability.
n) I do not believe AI scribe would save me time.
o) I had a negative experience using similar technology in the past, so I do not believe the AI scribe will be helpful.
p) I have concerns about patient privacy or data security.
q) I am waiting to hear feedback from others before deciding.
r) My team/manager has not endorsed or promoted its use.
s) There was no local clinical champion promoting or supporting its use in my area.
t) I feel uneasy with new tools and approaches.

## Section 3: Open-Ended Questions

Please answer these questions in relation to the AI scribe:

1. Were there any other barriers or concerns that have impacted your use of the AI scribe? (open text)
2. Is there anything that would help you want to use AI scribe? (open text)

**Would you be willing to participate in a short interview to discuss your reasons for not using the AI scribe?**

☐ Yes -> Please enter your email address below so a member of the research team can contact you: ______________________________

☐ No

*******Trained/Untrained Adopters Pathway*******

## Section 2: Perceived ease of use

1. **On a typical week, how often would you use the AI scribe?**

- Occasionally (with a few patients)
- With some (under half) of my patients
- With most (over half) of my patients
- With all my patients
  2. **Instructions:** For each statement below, please indicate your level of agreement in relation to the AI Scribe:

1 = Strongly Disagree, 2 = Disagree, 3 = Neutral, 4 = Agree, 5 = Strongly Agree

a) I am confident in my understanding of what an AI scribe is.
b) I am confident in my understanding of how my documentation is stored when using the AI scribe.
c) Learning to use the AI scribe was easy for me.
d) I quickly became comfortable using the AI scribe.
e) Editing AI-generated notes is simple.
f) The training prepared me to use the AI scribe effectively.
g) I was easily able to customise the AI scribe to suit my needs.
h) Explaining the AI scribe to patients and obtaining their consent is straightforward and easy.
i) The AI scribe struggled with specialised discipline terminology.
j) The AI scribe had difficulty capturing certain types of patient encounters.
k) I feel confident in my ability to use the AI scribe independently.
l) The AI scribe is difficult to use during a busy clinical shift.
m) I am unable to use the AI scribe as I do not have access to the equipment.
n) The AI scribe fits well into my existing documentation workflows.
o) I have access to the right environment (e.g., private room) to use the AI scribe.
p) I am not able to integrate the AI scribe as much as I had hoped, as patients decline to use it.
q) I had access to adequate support and guidance while using the AI scribe.
r) My manager encouraged me to try the AI scribe.
s) My colleagues are supportive of using the AI scribe.
t) I found it difficult to speak freely when the AI scribe was active.
u) I had concerns about patient privacy while using the AI scribe.
v) I would prefer some consultations not to be recorded by the AI scribe.
w) Using the AI scribe is easier than documenting my notes manually.

**Section 3: Perceived usefulness**

**1. Instructions:** For each statement below, please indicate your level of agreement in relation to the AI scribe:

1 = Strongly Disagree, 2 = Disagree, 3 = Neutral, 4 = Agree, 5 = Strongly Agree

a) The AI scribe helped improve the accuracy of my notes.
b) The AI scribe helped improve the organisation and/or structure of my notes.
c) The AI scribe helped improve the language and/or expression of my notes.
d) I felt that managing the AI scribe required too much of my cognitive attention during clinical work.
e) The AI scribe captures all relevant details in the consultation.
f) The AI scribe supported team-based care.
g) The AI scribe helped me save time when completing documentation.
h) The AI scribe allowed me to give patients more time.
i) The AI scribe made documentation feel more burdensome.
j) Patients appeared comfortable with me using the AI scribe.
k) The AI scribe improved my clinical workflow.
l) The AI scribe allowed me to focus more on the patient.
m) I am uncomfortable with how the AI scribe handles sensitive information.
n) The AI scribe reduced my stress related to documentation.
o) The presence of the AI scribe limited the usefulness of the consultation for certain sensitive topics.
p) I felt distracted or unsettled when the AI scribe was active during consultations.

## Section 4: Overall Experience & Impact

1. **Instructions:** For each statement below, please indicate your level of agreement in relation to the AI scribe:

1 = Strongly Disagree, 2 = Disagree, 3 = Neutral, 4 = Agree, 5 = Strongly Agree

## a) Overall, the AI scribe made my job more satisfying.

## b) Overall, the AI Scribe improved the interaction with the patient.

## c) Overall, the AI scribe enhanced patient care.

## d) Overall, I did not enjoy using the AI scribe.

## e) Overall, the AI scribe improved the quality of my documentation.

## f) Overall, the AI scribe enhanced the flow and efficiency of my clinical work.

## g) Overall, I have a positive attitude toward the AI scribe in clinical settings.

## h) Overall, I would recommend the AI scribe to other clinicians.

## i) Overall, I would like to continue using the AI scribe in the future.

## Section 5: Open-Ended Questions

Please answer these questions in relation to the AI scribe**:**

1. What was the most helpful aspect of using the AI scribe? *(open text)*
2. What challenges or limitations did you experience using the AI scribe? *(open text)*
3. What barriers were there to using the AI scribe? *(open text)*
4. What made using the AI scribe easier? *(open text)*
5. Do you have suggestions for improving the AI scribe or its ongoing implementation? *(open text)*

**Would you be willing to participate in a short interview to discuss your experiences with and opinions of the AI scribe?**

☐ Yes - Please enter your email address below so a member of the research team can contact you: ______________________________

☐ No
