# Supplementary material for: Determinants of AI Scribe Adoption in Allied Health: Mixed Methods Study Guided by the Capability, Opportunity, and Motivation Model for Behavior Change
Source: J Med Internet Res. 2026 Oct 15;28:e88088. doi: 10.2196/88088 (PMC13670898; doi:10.2196/88088)
Supplement: Supplementary file 3 [file jmir-v28-e88088-s002.docx]

**Clinician Interview Guide: AI Medical Scribe (Adopters)**

**Introduction**

Thank you for taking the time to participate in this interview. We want to understand your experience using the AI scribe and how it impacted your documentation process and patient care. Your feedback will help us assess the benefits and challenges of integrating AI into clinical documentation.

Do you agree to having this interview recorded?

1. **General Experiences**

**1.1 Overall, how was your experience using the AI scribe?**

Prompts:

- How long have you been using it for?
- How often do you use it, and in what contexts?
- How did it compare to your usual documentation process?
- Do you use it for letters/iEMR power forms/consult notes?

**1.2 How did the AI scribe impact patient care?**

Prompts:

- Did it impact communication or rapport with patients?
- Did it affect the amount of time you spent with patients?
- Did it create distractions during consultations?
- Did you change your approach or manner due to the AI scribe?

**1.3 How did the AI scribe affect your workload?**

Prompts:

- Did using the AI scribe influence how you managed your daily workflow? If so, how?
- What impact, if any, did it have on your efficiency or pace of work?

**1.4 How did the AI scribe impact your satisfaction in your role?**

Prompts:

- Did it influence your enjoyment in your work?
- Did it affect your stress levels?

**2. Ease of Use and Implementation**

**2.1 How difficult or easy was it to use the AI scribe?**

**Prompts:**

- Do you now feel confident using it? If so, how long did that take?
- What helped or would help build your confidence?
- Do you understand the AI scribe and how the data is stored?
- How confident do you feel in explaining the scribe to patients and obtaining consent?

**2.2 How easy was it to customise the AI scribe for your needs?**

Prompts:

- Were you able to adjust the AI scribe settings/customise it to suit your needs for your role?
- How difficult or easy was the customisation process?

**2.3 What barriers, if any, did you encounter when using the AI scribe?**

Prompts:

- Did you experience any technological barriers (e.g., equipment or software issues)?
- Did you experience any environmental barriers (e.g., room setup or noise)?
- Did you encounter any contextual barriers related to specific patient circumstances, presentations, or patient groups?
- Did workflow factors, such as high-pressure situations or fast-paced requirements, present any challenges?
- Did you find it difficult to remember to use the AI scribe or to integrate it into your routine?

**2.4 What factors, if any, supported your use of the AI scribe?**

Prompts:

- Did the training provide sufficient preparation?
- Were there any other tools or resources that supported the use of the AI scribe?
- Did you receive support from peers or managers? If so, did it make a difference? If not, do you think it would have been helpful?
- Did your previous experience with AI or similar digital tools influence how you used the AI scribe? If so, how?

**3. Usefulness and Impact**

**3.1 Can you describe situations where the AI scribe was useful?**

Prompt:

- Did it address challenges with traditional note-taking?

**3.2 Can you describe situations where it was not useful?**

Prompt:

- Were there any situations or specific patient groups where you found the AI scribe to be less useful or potentially unsuitable?

**3.3 What are your views on the quality and accuracy of the AI scribe?**

**Prompts:**

- Did using the AI scribe have any impact on the accuracy of your documentation? If so, how (e.g., level of detail)?
- Did using the AI scribe affect the quality of your documentation in any way (e.g. clarity, or structure)?

**3.4 How well did the AI scribe align with the needs of your role in allied health?**

- To what extent did the AI scribe capture information relevant to your role during the conversation?
- How suitable was the way the AI scribe presented the information for your role?
- How effectively did the AI scribe handle any specialised terminology used in your practice?

**3.5 What features would make the AI scribe more useful for you?**

Prompts:

- Are there features or improvements you would like to see?
- Would more automation, customisation, or integration help?

**4. Future Use and Overall Perception**

**4.1 Do you intend to continue using the AI scribe in the future?**

Prompts:

- Why or why not?
- What factors would influence this decision?
- Would you recommend using the AI scribe to colleagues?

**4.2 Do you have any final thoughts or suggestions for improving the AI scribe in clinical practice?**
