# Supplementary material for: Determinants of AI Scribe Adoption in Allied Health: Mixed Methods Study Guided by the Capability, Opportunity, and Motivation Model for Behavior Change
Source: J Med Internet Res. 2026 Oct 15;28:e88088. doi: 10.2196/88088 (PMC13670898; doi:10.2196/88088)
Supplement: Supplementary file 4 [file jmir-v28-e88088-s003.docx]

**Interview Guide: Non-adopter clinicians**

Thank you for taking the time to participate in this interview. We are interested in understanding why some eligible clinicians have not yet signed up to try the AI Scribe. Your perspectives will help us identify what might support or hinder adoption and improve how the tool is introduced.

Are you happy for me to record?

**1. Broad Impressions and Barriers**

1.1 What are your initial thoughts or feelings about the AI scribe?
1.2 What has been the biggest barrier to signing up or trying it?
1.3 If you have signed up/completed the training, how long ago was this?
1.4 Have you previously used Dragon Medical One? If so, how do you feel the AI scribe differs from this technology?
1.5 How do you feel about using new technology in clinical practice generally?

**2. Understanding Awareness and Capability**

2.1 Can you explain what knowledge or skills are needed to use a tool like the AI scribe?

Prompt

- Do you feel you understand what the AI scribe does and how it works?
- Are you aware of the Learning Online (LOL) training?
- If you have completed the Learning Online (LOL) training, how do you feel this prepared you to use the AI scribe?
- Was there anything else which could have helped prepare you for using the AI scribe?

**3. Environmental and Social Context**

3.1 Were there any other barriers which prevented you from using the AI scribe?

- Any environmental/technical barriers (e.g., access to a private room or device)?
- Any organisational barriers?

3.2 Has time or opportunity impacted you being able to use the AI scribe or undertake the training?

3.3 Was the AI scribe discussed or promoted within your team or by leadership?

**4. Attitudes and Beliefs**

4.1 Do you believe an AI scribe could be useful in your clinical work? Why or why not?

4.2 Do you have any concerns about trying it?

**Prompt:**

- Do you have concerns around privacy?
- Do you have concerns about accuracy?
- Do you have concerns about patient care?
- Do you have concerns about workflow disruption?

**5. Future Considerations**

5.1 What would help or encourage you to consider using the AI scribe in the future?
