# Supplementary material for: Determinants of AI Scribe Adoption in Allied Health: Mixed Methods Study Guided by the Capability, Opportunity, and Motivation Model for Behavior Change
Source: J Med Internet Res. 2026 Oct 15;28:e88088. doi: 10.2196/88088 (PMC13670898; doi:10.2196/88088)
Supplement: Supplementary file 5 [file jmir-v28-e88088-s004.docx]

Appendix Figure 4.1: Adopter Survey Results to Capability Questions

Adopter Survey Results to Capability Questions (n=58)

|  | Strongly Disagree | Disagree | Neutral | Agree | Strongly Agree |
| --- | --- | --- | --- | --- | --- |
| The training prepared me to use the AI scribe effectively | 0 | 12.1 | 19 | 48.3 | 20.7 |
| I am confident in my understanding of how my documentation is stored when using the AI scribe | 1.7 | 3.4 | 3.4 | 48.3 | 43.1 |
| I am confident in my understanding of what an AI scribe is | 1.7 | 0 | 0 | 46.6 | 51.7 |

Appendix Figure 4.2: Adopter Survey Results to Opportunity Questions

Adopter Survey Results to Opportunity Questions (n=58)

|  | Strongly Disagree | Disagree | Neutral | Agree | Strongly Agree |
| --- | --- | --- | --- | --- | --- |
| Explaining the AI scribe to patients and obtaining their consent is straightforward and easy | 0 | 3.4 | 12.1 | 37.9 | 46.6 |
| I am unable to use the AI scribe as I do not have access to the equipment | 65.5 | 25.9 | 5.2 | 1.7 | 1.7 |
| The AI scribe fits well into my existing documentation workflows | 3.4 | 8.6 | 12.1 | 44.8 | 31 |
| I have access to the right environment (e.g., private room) to use the AI Scribe | 3.4 | 5.2 | 10.3 | 32.8 | 48.3 |
| I am not able to integrate the AI scribe as much as I had hoped, as patients decline to use it | 51.7 | 34.5 | 6.9 | 0 | 6.9 |
| I had access to adequate support and guidance while using the AI scribe | 0 | 12.1 | 19 | 46.6 | 22.4 |
| My manager encouraged me to try the AI scribe | 3.4 | 8.6 | 24.1 | 34.5 | 29.3 |
| My colleagues are supportive of using the AI scribe | 0 | 0 | 13.8 | 46.6 | 39.7 |

Appendix Figure 4.3: Adopter Survey Results to Motivation Questions

Adopter Survey Results to Motivation Questions (n=58)

|  | Strongly Disagree | Disagree | Neutral | Agree | Strongly Agree |
| --- | --- | --- | --- | --- | --- |
| Using the AI scribe is easier than documenting my notes manually | 6.9 | 10.3 | 8.6 | 32.8 |  |
| I would prefer some consultations not to be recorded by the AI scribe | 32.8 | 31 | 13.8 | 22.4 |  |
| I had concerns about patient privacy while using the AI scribe | 43.1 | 50 | 3.4 | 0 | 3.4 |
| I found it difficult to speak freely when the AI scribe was active | 46.6 | 48.6 | 1.7 | 1.7 | 1.7 |
